# Initial Outcomes of a Novel Technique of Nipple Sparing Mastectomy Without Reconstruction

**DOI:** 10.3390/cancers17060984

**Published:** 2025-03-14

**Authors:** Geok Hoon Lim, Nathalie Liew, John Carson Allen

**Affiliations:** 1 Breast Department, KK Women’s and Children’s Hospital, 100 Bukit Timah Road, Singapore 229899, Singapore; nathalie.liew.y.h@singhealth.com.sg; 2 Duke-NUS Medical School, 8 College Rd., Singapore 169857, Singapore

**Keywords:** breast cancer, nipple sparing mastectomy, breast reconstruction, minimal scar mastectomy, nipple areola complex necrosis

## Abstract

**Introduction:** Nipple sparing mastectomy is oncologically safe and has a good cosmetic outcome. However, nipple sparing mastectomy was conventionally performed with reconstruction. Minimal scar mastectomy (MSM) is a novel technique which could allow women, with non-ptotic breasts, who do not want reconstruction, to conserve their nipple areolar complex (NAC) and avoid the transverse scar associated with modified radical mastectomy. This is the first study on the outcomes of MSM. **Methods**: MSM complications and their risk factors, recurrence rates and cosmetic outcomes were assessed. As MSM is a modification of the round block technique, the mean ring distance (MRD), which is the average of the distance between the inner and outer ring circumferentially, was assessed, too. **Results:** A total of 28 patients (29 breasts) were analysed. There was no recurrence after a mean/median follow-up of 40.3/41 months (4–80 months). In the initial recruitment of 17 patients (18 breasts), NAC necrosis occurred in eight cases (three complete, five partial). Prediabetes (*p* = 0.0128) and MRD ≥1.5 cm (*p* = 0.0440) were statistically significant for NAC necrosis. Of the available data, 11/15 (73.3%) rated the cosmetic outcome as excellent/good, with poorer cosmetic outcome correlated with NAC necrosis (*p* = 0.006). Avoiding the above risk factors in the next 11 patients, NAC necrosis decreased to 1/11 (9.0%) with mild ischaemia. Cosmetic outcome was rated as excellent/good in 90.9%. **Conclusions:** MSM is oncologically safe and is best performed in patients with no risk factors for NAC necrosis, including prediabetes and MRD < 1.5 cm. These pilot results will refine the selection criteria of patients for MSM.

## Simple Summary

Nipple sparing mastectomy was conventionally performed with reconstruction. Minimal scar mastectomy (MSM) is a novel technique which could allow women, with non-ptotic breasts, who do not want reconstruction, to also conserve their nipple and avoid the transverse scar associated with mastectomy and no reconstruction. This is the first study on the initial oncologic and surgical outcomes of MSM. MSM was oncologically safe with no recurrence after a median follow-up of 41 months. Nipple necrosis was more likely in patients with risk factors including prediabetes and with a greater amount of breast skin for reduction during mastectomy, measured as MRD (mean ring distance) ≥1.5 cm. Excluding the cases with risk factors, nipple necrosis was 9%, which is compatible with the literature. The majority rated the cosmetic outcome as excellent/good. These pilot results will refine the selection criteria of patients for MSM.

## 1. Background

In breast cancer patients without nipple areolar complex (NAC) involvement, a nipple sparing mastectomy can be performed and has been shown to be oncologically safe [1,2]. However, nipple sparing mastectomy has conventionally been performed with breast reconstruction.

**Figure 1:**
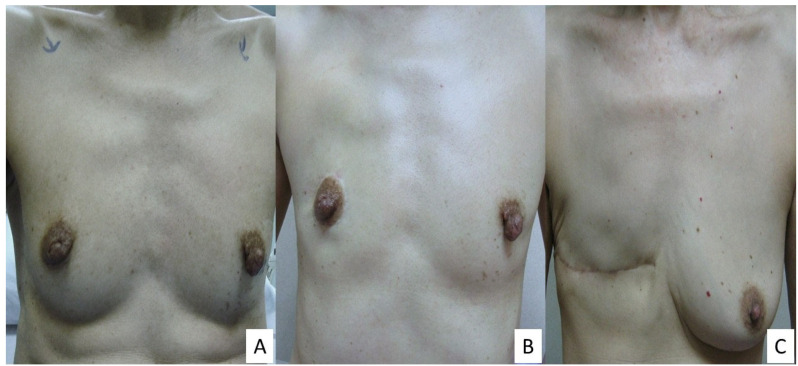
(**A**) Preoperative photo of patient. (**B**) Photo of patient 18 months after right MSM in contrast to. (**C**) Another patient with a right modified radical mastectomy.

In patients with non-ptotic breasts, a novel technique, known as minimal scar mastectomy (MSM), could be performed, allowing suitable patients to conserve their NAC during a mastectomy without undergoing a reconstruction [3]. In contrast to the transverse chest scar in a modified radical mastectomy, MSM not only allows the preservation of NAC, but results in a well-concealed scar around the areola (Figure 1), giving a better cosmetic outcome.

While this novel technique was previously described, its feasibility and safety have not been evaluated. We aim to evaluate the initial oncological and surgical outcomes of MSM and further refine the selection criteria of patients most suitable for MSM. This is the first study reporting on the outcomes of MSM.

## 2. Materials and Methods

Patients who were diagnosed with breast cancer and underwent MSM from 1 March 2017 to 31 May 2024 at KK Women’s and Children’s Hospital, Republic of Singapore, were analysed from a prospectively kept database. Patients who were offered MSM must have little breast ptosis as an inclusion criterion. These patients were offered the option of breast conservation if they were suitable candidates or mastectomy with or without reconstruction. If the patient had decided for mastectomy and did not wish to undergo reconstruction, the option of minimal scar mastectomy versus conventional modified radical mastectomy was discussed. Patients who were excluded from MSM were patients with known diabetes, smokers, NAC or extensive skin involvement, inflammatory breast cancer and patients with moderate to severe breast ptosis.

All patients received subsequent treatment based on the recommendations from the multidisciplinary meeting.

The clinical, radiological and pathological features of these patients were obtained and reviewed. For invasive cancer, tumour size was measured based on the invasive cancer size. In patients after neoadjuvant chemotherapy, the tumour size was measured based on the residual pathological invasive cancer/in situ cancer size. For patients who underwent completion mastectomy due to involved margins from lumpectomy and patients with multiple cancers, the tumour size was based on the largest reported invasive cancer size.

The oncological safety of MSM, analysed in terms of any recurrence, and post-surgical complications such as haematoma, nipple necrosis rates, etc., were reviewed. The cosmetic outcome of MSM was assessed by the patients using a score of 1–4 with 1 being excellent, 2 good, 3 fair and 4 poor.

During the study, the patients who were recruited in the initial phase of the study from 1 March 2017 to 30 April 2021 were analysed for interim outcomes. The findings from the interim analysis were then adopted in the selection of patients for the latter part of the study. The findings from the patients included in the latter part of the study were reported, too.

### 2.1. Statistical Analysis

Continuous variables were summarised as mean (range) and categorical variables as frequency counts and percentages. Frequency count (%) distributions of clinical, radiological and pathological features between patients who underwent MSM with no NAC necrosis versus those with partial/complete NAC necrosis were statistically compared using Fisher’s exact test. The clinical, radiological and pathological features were also compared between patients with self-rated excellent/good cosmetic outcomes versus fair/poor cosmetic outcomes using Fisher’s exact test. All statistical analysis was performed using SAS v9.4 software (SAS Inc., Cary, NC, USA).

### 2.2. Surgical Technique of MSM

**Figure 2:**
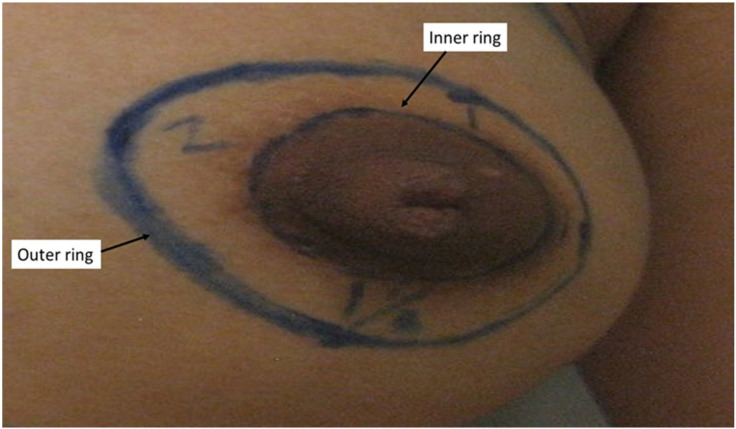
Photo showing the distance between the inner and outer ring. In this case, it was 1 cm, 1 cm, 1.5 cm and 2 cm at the superior, lateral, inferior and medial aspect. The mean ring distance (MRD) was (1 + 1 + 1.5 + 2)/4 = 1.375 cm. A MRD < 1.5 cm was associated with a low risk of NAC necrosis.

The surgical steps of MSM were previously described [3]. Firstly, the excess skin that needs to be removed following the breast volume loss in the mastectomy was estimated [4] and drawn as an outer ring around the areola. The inner ring was formed by the outline of the areola. The distance between the inner and outer ring was measured at the superior, lateral, inferior and medial aspect (Figure 2) in centimetres. The average of these 4 measurements was calculated for the breast and denoted as the mean ring distance (MRD). MRD signifies the amount of skin to be reduced during the mastectomy.

This excess skin was then de-epithelised between the outer and inner ring, like what was performed in an oncoplastic round block technique [5]. A full thickness incision was then made along the de-epithelised area, not exceeding more than half the circumference of the outer ring. Meanwhile, the NAC was raised and supported on a vascular pedicle. Mastectomy was then performed via the full thickness incision.

A purse-string of the outer ring was next performed to achieve the diameter of the contralateral NAC. Finally, the outer and inner ring were sutured together, giving the final circum-areolar scar.

This study obtained approval from SingHealth Centralised Institutional Review Board (CIRB Ref: 2020/2147). Informed consent from the patients were obtained.

This study was registered on ClinicalTrials.gov Identifier NCT06676761. In addition, it has been reported in line with the STROCSS criteria [6].

## 3. Results

A total of 29 patients (30 breasts) underwent MSM. One patient had bilateral MSM. One patient was diagnosed with NAC involvement on the frozen section intra-operatively and had NAC removed. She was hence excluded from analysis. After exclusion of this patient, 28 patients (29 breasts) were available for analysis. Of these patients, 17 patients (18 breasts) were recruited in the initial phase of the study from 1 March 2017 to 30 April 2021 while the remaining patients in the latter part of the study were included after excluding the risk factors for NAC necrosis, which were found in the initial phase of the study.

For the 17 patients (18 breasts) recruited in the initial phase of the study, the mean age of the patients was 57 years old (range: 36–72). The mean body mass index (BMI) was 21.4 kg/m^2^ (range: 17.1–27.9) (Table 1). There was one patient with connective tissue disease, one patient had breast cancer recurrence after prior lumpectomy and radiotherapy and four patients with prediabetes. There were no smokers. All had no or grade 1 breast ptosis. The average sternal notch to nipple distance was 19.7 cm (range: 17.5–23). The average MRD was 1.67 cm (range: 1–2.5). The minimum and maximum distance between the inner and outer ring was recorded as 0.5 cm and 3 cm, respectively.

Based on ultrasound imaging, the mean distance of nipple to cancer was 2.7 cm (range: 1–5). Five cases also had multifocal/multicentric cancers/ lesions within the breast which required excision.

Two patients underwent neoadjuvant chemotherapy. Two other patients had involved margins from lumpectomy and underwent MSM for completion mastectomy. Histologically, 13 cases had invasive ductal carcinoma, 2 cases had ductal carcinoma in situ and the remaining 3 cases had mucinous carcinoma, invasive tubular carcinoma and mixed invasive ductal and lobular carcinoma respectively. Five, six and seven cases were grade I, II and III cancers, respectively. The mean tumour size was 16.2 mm (range: 0.9–49). Only one patient had N2 disease, two patients had nodal pathological complete response after neoadjuvant chemotherapy and the rest of the patients had N0 disease. A total of 83.3%, 72.2% and 33.3% were positive for oestrogen receptors, progesterone receptors and human epidermal growth factor receptors 2 (HER2), respectively.

The mean mastectomy weight was 334.5 g (range: 79–765 g). Three cases (16.7%) had smaller breasts than the contralateral breast due to previous ipsilateral breast surgery.

A total of 10 cases did not have NAC necrosis. NAC necrosis was, however, noted in eight cases. Three and five cases had complete and partial peripheral NAC necrosis, respectively. In all these cases, the NAC necrosis was treated conservatively. There were no other complications.

Four out of seventeen (23.5%) patients received adjuvant chemotherapy. Four out of eighteen cases (22.2%) received adjuvant radiotherapy, of which only one developed partial NAC necrosis. There was, however, no delay in starting adjuvant treatment.

Of the eight cases which had NAC necrosis, four cases had prediabetes prior to operation and one case had connective tissue disease. Another case had periareolar skin involvement, which compromised NAC vascularity. Excluding these six cases of NAC necrosis with pre-disposing risk factors and another case with predisposing risk factor but which did not have NAC necrosis, the necrosis rate for the series of 11 breast cases without known predisposing risk factors was 2/11 (18.2%).

Of those patients who were assessed, 11/15 (73.3%) rated the cosmetic outcome as excellent or good, while 4/15 rated it as fair. These patients who rated it as fair had complete or partial NAC necrosis.

Statistical analysis revealed significant correlation between risk factors for NAC necrosis (*p* = 0.0128) and MRD ≥ 1.5 cm (*p* = 0.0440) with NAC necrosis. Patients with NAC necrosis also reported a statistically significant poorer cosmetic outcome (*p* = 0.006). The remaining parameters did not report any statistically significant relationship with NAC necrosis or cosmetic outcome.

Based on the findings from the interim analysis of these 17 patients (18 breasts) recruited in the initial phase of the study, the subsequent 11 patients recruited, avoiding the above risk factors, were analysed.

The mean age of these 11 patients was 52.7 years old (range: 40–65). The mean body mass index (BMI) was 20.9 kg/m^2^ (range: 18.0–26.2). All had no or grade 1 breast ptosis. The average MRD was 1.18 cm (range: 0.75–1.45). None had known risk factors for NAC necrosis. Three out of eleven cases (27.3%) received adjuvant radiotherapy and none developed NAC necrosis. The mean mastectomy weight was 178.7 g (range: 50–341 g).

The pathological characteristics (Table 2) were similar to the patients recruited initially in the study with the mean tumour size of 15.7 mm (range: 3–50).

Of these 11 cases, only 1 patient (9.1%) developed mild NAC necrosis, which was treated conservatively. Ten out of eleven (90.9%) rated the cosmetic outcome as excellent or good.

For all the 29 cases, there was no recurrence after a mean/median follow up of 40.3/41 months (4–80 months).

## 4. Discussion

MSM is oncologically safe based on a mean follow-up of 40.3 months. It is suitable for patients with minimal ptosis undergoing a mastectomy and who desire to preserve their NAC but do not want reconstruction. It was best performed in patients without risk factors for NAC necrosis, including prediabetes and MRD < 1.5, to achieve a good postoperative and cosmetic outcome. By avoiding the above risk factors, the NAC rate decreased to 9.1%. This is the first reported study on the outcomes of MSM and these results can aid in the selection of suitable patients for MSM to achieve the best outcome.

In carefully selected patients, nipple sparing mastectomy is oncologically safe with low reported recurrence rates of 0–7.2% [7]. Compared to a skin sparing mastectomy, preservation of the NAC results in a better cosmetic outcome [8]. Though nipple sensation may not be preserved all the time [9], nipple sparing mastectomy has still been associated with improved psychological well-being [10]. However, nipple sparing mastectomy is conventionally performed in conjunction with breast reconstruction.

As a result, in patients who had to undergo a mastectomy but did not desire reconstruction, the NAC could not be preserved. These patients would undergo a modified radical mastectomy, leaving a transverse chest scar. MSM is a newly described technique which allows patients who need a mastectomy but do not desire a breast reconstruction an option to preserve their NAC and provide a well concealed scar. This technique uses the round block principle, which has shown to have comparable operative parameters as standard wide local excision [11]. This technique is, however, applicable only to women with no/minimal ptosis of the breast. In this study, MSM has been shown to be oncologically safe, like nipple sparing mastectomy, since the same oncologic inclusion criteria for patients with nipple sparing mastectomy were applied as well.

NAC necrosis has been associated with multiple risk factors such as smoking, diabetes [12] and type of incision placement. Of all the various types of incision placement, the periareolar incision was associated with the highest risk of NAC necrosis [13,14] because of the disruption of the NAC blood supply. The NAC necrosis rate associated with periareolar incision had been reported to be on average 18.10% [7] and could be as high as 38.1% [15]. In a paper by Ahn SJ et al., the nipple ischemia rate in nipple sparing mastectomy has been cited as high as 64.1% [13]. After excluding patients with risk factors for NAC necrosis in the latter part of our study, our NAC necrosis rate of 9.1% was not worse compared with the reported periareolar incision-associated necrosis rate. The periareolar incision needs to be used in MSM to centralise the NAC following the mastectomy and to conceal the scar.

As the periareolar incision was associated with the highest risk of NAC necrosis among all the incisions used, it is important that the patients do not have other contributing risk factors. While diabetics is a known risk factor for NAC necrosis, the relevance of prediabetes as a risk factor for NAC necrosis is not known, though prediabetes has been implicated with a higher risk of peripheral artery disease [16] and mortality [17]. It is characterised by an elevated blood glucose level, which is not raised enough to be defined as having diabetes. It occurs when there is a fasting glucose level of 100 to 125 mg/dL, or a glycated hemoglobin level (HbA1C) measuring 5.7% to 6.4%. In our series, the patients with NAC necrosis were mostly patients with prediabetes; hence, this group of patients needs to be excluded when doing MSM.

To reduce the risk of NAC necrosis, measures such as a nipple delay [18] could be performed, especially in high-risk patients. Various techniques for nipple delay have been published [19], describing the undermining of the NAC and mastectomy flaps to varying degrees. Nipple delay also has the added benefit of allowing a biopsy of the NAC prior to the definitive mastectomy, avoiding false negative frozen section results of the retroareolar tissue, which can occur in 9.3% [20]. By performing a nipple delay, the risk of NAC ischemia could be reduced from 33% in the non-nipple delay group to 12% with nipple delay [21] However, since nipple delay constitutes another invasive procedure, this was not performed in our series.

In addition, intraoperative indocyanine green (ICG) fluorescence imaging could be used to predict the viability of the NAC and mastectomy flaps with 98.5–100% specificity and 83.8–100% sensitivity [22,23]. This was, however, not undertaken in our series since ICG has been also shown to have false positive results [24] and ultimately, clinical judgement needs to be exercised when interpreting the results of ICG.

In cases when there were threatened skin flaps following nipple sparing mastectomy, hyperbaric oxygen therapy is a promising therapy which could increase tissue oxygenation and vascularization, leading to improved wound healing [25]. In a series, flap salvage could be achieved in 88% of the cases [26]. However, more studies are needed to assess its long-term effects, cost effectiveness and ease of access.

While increased mastectomy weight and severe breast ptosis had been implicated as risk factors for NAC necrosis [27], this was, however, not demonstrated in our series since we have confined MSM to patients with no/minimal ptosis and relatively small breast size. On the other hand, MRD, a measure of the amount of skin to be reduced during mastectomy was statistically significant for NAC necrosis. This is a unique parameter only measured in MSM, and a greater average circumferential distance of the outer ring from the inner ring, resulting in MRD >/= 1.5 cm, could lead to a higher risk of NAC necrosis. This finding could be explained that with a greater amount of skin which needs to be reduced, the breast may be of a relatively larger volume or/and there is more lax skin, resulting in a longer NAC pedicle, hence compromising the NAC blood supply. In patients undergoing MSM, MRD may be a more indicative factor for NAC necrosis than mastectomy weight since it was reported that only mastectomy weight exceeding 400 g was significant for NAC necrosis [28]. This hypothesis awaits further investigation.

Radiotherapy after nipple sparing mastectomy could increase the risk of complications and affect cosmetic outcomes. This effect was most marked in patients with implant reconstruction [29]. Radiotherapy could also result in a higher risk of skin flap necrosis after nipple sparing mastectomy, though it did not seem to increase the risk of NAC necrosis, as reported in a systematic review [30]. This finding was also demonstrated in our series.

It was not surprising that the cosmetic outcome was related to NAC necrosis. This finding was like other studies [31]. In addition, in patients with smaller breast volume, the asymmetry noted after MSM may be less marked and could be postulated to result in a better cosmetic score, compared to those with a larger breast volume.

MSM could be particularly useful in the Asian population, who had experienced a surge in breast cancer cases [32]. Asian countries also have low reported screening rates [33], which resulted in a larger tumour size at presentation. Coupled with relatively smaller breast sizes in Asian women [34] with a high incidence of no breast ptosis [35], breast conservation could be difficult to achieve, resulting in higher reported mastectomy rates [36] than their Western counterparts. Rates of reconstruction after mastectomy also tended to be low in Asian patients [37]. In patients with early breast cancer, the rate of mastectomy without reconstruction, in some parts of Asia, has been reported to be as high as 81.7% [38]. Despite this, patients with mastectomy alone without reconstruction could be satisfied with their surgical outcomes [39].

Strengths of the paper included this being the first paper to describe the initial results of a novel technique, which could allow patients with mastectomy but desired no reconstruction to conserve their NAC and have a better cosmetic outcome than a modified radical mastectomy.

Limitation of this paper included a small sample size. This was, however, planned to be a pilot study to determine the selection criteria of patients most suitable for the novel technique of MSM. Though this was a small study, there were important findings, such as exclusion of patients with prediabetes and with MRD >/= 1.5 cm, to improve the NAC necrosis rate. Being a novel technique, MSM had a high nipple necrosis rate initially. This could be explained by a learning curve and not well-defined patient selection criteria initially. Selection criteria of MSM initially were based on the criteria for conventional nipple sparing mastectomy; however, it was subsequently demonstrated that there are additional factors such as prediabetes and large skin reduction to avoid, too. Another limitation was that there were other parameters, such as the length of operating time and the rate of preservation of the nipple sensation after MSM etc, which were not assessed. A larger prospective study, after incorporation of the initial lessons from this paper, could be planned to further validate the usefulness of the novel technique of MSM. In addition, the quality of life/satisfaction outcomes could be studied then and compared to a matched conventional mastectomy control group, using a validated questionnaire.

## 5. Conclusions

MSM is oncologically safe and is best reserved for patients with non-ptotic breasts with minimal skin reduction during mastectomy and no predisposing risk factors for NAC necrosis, including prediabetes, to obtain the best postoperative outcome. Based on these initial results, larger prospective studies could be planned to further validate this novel technique.

## Figures and Tables

**Table 1:** Clinical, radiological and pathological features of patients who underwent MSM in the initial part of the study. BMI—body mass index, NAC—nipple areolar complex. MRD—mean ring distance. HER2—human epidermal growth factor receptor 2.

	Cases with no NAC Necrosis N = 10/(%)	Cases with Partial/Complete NAC Necrosis N = 8/(%)	*p* Value
Clinical			
Age/years			0.3137
<50	4 (40)	1 (12.5)	
≥50	6 (60)	7 (87.5)	
BMI (kg/m^2^)			0.2986
<18.5	2 (20)	3 (37.5)	
18.5–22.9	3 (30)	0 (0)	
23–26.9	5 (50)	5 (62.5)	
>27	0 (0)	0 (0)	
Sternal notch to NAC distance/cm			0.5227
≤20	6 (85.7)	3 (60)	
>20	1 (14.3)	2 (40)	
Missing	3	3	
Risk factors for NAC necrosis			0.0128
Yes	1 (10)	6 (75)	
No	9 (90)	2 (25)	
MRD/cm			0.0440
<1.5	5 (55.6)	0 (0)	
≥1.5	4 (44.4)	6 (100)	
Missing	1	2	
Cosmetic score			0.0060
Excellent	5 (50)	0 (0)	
Good	5 (50)	1 (20)	
Fair	0 (0)	4 (80)	
Poor	0 (0)	0 (0)	
Missing	0 (0)	3	
Radiological			
Distance of tumour from NAC/cm			0.3469
≤2	3 (33.3)	5 (62.5)	
>2	6 (66.7)	3 (37.5)	
Missing	1	0	
Multifocality			0.6078
Yes	2 (20)	3 (37.5)	
No	8 (80)	5 (62.5)	
Pathological			
Histology			0.5882
Ductal carcinoma in situ	2 (20)	0 (0)	
Invasive ductal cancer	6 (60)	7 (87.5)	
Others	2 (20)	1 (12.5)	
Tumour size/mm			1.0000
≤20	7 (70)	5 (62.5)	
21–50	3 (30)	3 (37.5)	
>50	0 (0)	0 (0)	
Nodal status			1.0000
N0	8 (80)	7 (87.5)	
N1	0 (0)	0 (0)	
N2	1 (10)	0 (0)	
N3	0 (0)	0 (0)	
ypN0	1 (10)	1 (12.5)	
Grade			0.8400
I	2 (20)	3 (37.5)	
II	4 (40)	2 (25.0)	
III	4 (40)	3 (37.5)	
Estrogen receptor			0.5588
Positive	9 (90)	6 (75)	
Negative	1 (10)	2 (25)	
Progesterone receptor			1.0000
Positive	7 (87.5)	6 (75)	
Negative	1 (12.5)	2 (25)	
Missing	2	0 (0)	
HER2 receptor *			1.0000
Positive	3 (37.5)	3 (37.5)	
Negative	5 (62.5)	5 (62.5)	
Mastectomy weight/g			0.6339
≤100	1 (10)	0 (0)	
101–200	3 (30)	1 (12.5)	
201–300	3 (30)	1 (12.5)	
301–400	0 (0)	1 (12.5)	
401–500	2 (20)	3 (37.5)	
>501	1 (10)	2 (25)	

* for invasive cancers only.

**Table 2:** Clinical, radiological and pathological features of 11 patients who underwent MSM in the latter part of the study. BMI—body mass index, NAC—nipple areolar complex. MRD—mean ring distance. HER2—human epidermal growth factor receptor 2.

	Cases with no NAC Necrosis N = 10/(%)	Cases with Mild NAC Necrosis N = 1/(%)
Clinical		
Age/years		
<50	1 (10)	1 (100)
≥50	9 (90)	0 (0)
BMI (kg/m^2^)		
<18.5	1 (10)	0 (0)
18.5–22.9	7 (70)	1 (100)
23–26.9	2 (20)	0 (0)
Cosmetic score		
Excellent	4 (40)	0 (0)
Good	6 (60)	0 (0)
Fair	0 (0)	1 (100)
Poor	0 (0)	0 (0)
Radiological		
Distance of tumour from NAC/cm		
≤2	4 (40)	1 (100)
>2	6 (60)	0 (0)
Multifocality		
Yes	2 (20)	1 (100)
No	8 (80)	0 (0)
Pathological		
Histology		
Ductal carcinoma in situ	2 (20)	0 (0)
Invasive ductal cancer	8 (80)	1 (100)
Others	0 (0)	0 (0)
Tumour size/mm		
≤20	9 (90)	1 (100)
21–50	1 (10)	0 (0)
>50	0 (0)	0 (0)
Nodal status		
N0	7 (70)	1 (100)
N1	2 (20)	0 (0)
N2	0 (0)	0 (0)
N3	0 (0)	0 (0)
ypN1	1 (10)	0 (0)
Grade		
I	1 (10)	0 (0)
II	8 (80)	1 (100)
III	1 (10)	0 (0)
Estrogen receptor		
Positive	8 (80)	0 (0)
Negative	2 (20)	1 (100)
Progesterone receptor		
Positive	7 (70)	0 (0)
Negative	1 (10)	1 (100)
Missing	2 (20)	0 (0)
HER2 receptor *		
Positive	3 (37.5)	1 (100)
Negative	5 (62.5)	0 (0)
Mastectomy weight/g		
≤100	2 (20.0)	0 (0)
101–200	5 (50.0)	1 (100)
201–300	1 (10.0)	0 (0)
301–400	2 (20.0)	0 (0)

* for invasive cancers only.

## Data Availability

The datasets used and/or analysed during the current study are available from the corresponding author on reasonable request.
